# An autonomous navigation system with a trajectory prediction-based decision mechanism for rubber forest navigation

**DOI:** 10.1038/s41598-024-81084-9

**Published:** 2024-11-27

**Authors:** Xirui Zhang, Yongqi Liu, Junxiao Liu, Xuanli Chen, Ruiwu Xu, Weiqiang Ma, Zhifu Zhang, Shaohua Fu

**Affiliations:** 1 School of Mechanical and Electrical Engineering, Hainan University, Hainan, China; 2 School of Information and Communication Engineering, Hainan University, Hainan, China

**Keywords:** Rubber-tapping robot, Autonomous navigation system, Multi-objective navigation, Navigation decision mechanism, Electrical and electronic engineering, Computer science

## Abstract

The use of rubber-tapping robots capable of autonomous navigation in place of manual rubber-tapping is a growing trend, but the challenging multi-objective navigation task in forest environments impedes their autonomous operation. To tackle this issue, an autonomous navigation system with a trajectory prediction-based decision mechanism for rubber forest navigation is designed. This navigation decision mechanism is comprised of obtaining coordinates of target points (OCTP), selecting the next coordinate (SNC), generating the additional coordinates (GAC), and optimizing the planned paths (OPP). By utilizing this mechanism, the robot can autonomously select the next target point based on its current position and the actual operating logic while navigating in the forest areas, adding additional coordinates during row or column changes, and planning and optimizing the path. The on-site experiments demonstrate that during autonomous navigation, the positioning accuracy is favorable and supports subsequent operations. The overall rationality of the planned path reaches 92.14%, further confirming its effectiveness.

## Introduction

Natural rubber, being a crucial strategic and industrial material^1^, has faced various challenges in its production process. The acquisition of natural rubber has traditionally depended on manual rubber-tapping, a task characterized by high work intensity, low wages, and demanding technical skills^2^, all contributing to the increasing severity of the aging problem among rubber workers. In light of the rapid advancements in artificial intelligence and control engineering, the emergence of autonomous mobile robots has revolutionized various industries^3,4^. The development of rubber-tapping robots provides a promising solution to address the challenges faced by the natural rubber industry. A key aspect of this solution involves equipping rubber-tapping robots with the ability to autonomously navigate through rubber forests, thus enhancing efficiency and productivity in natural rubber production.

In the context of robot navigation technology, key components encompass environmental sensing, precise positioning, navigation decision, and path planning^5,6^. Autonomous navigation of robots relies heavily on environment sensing and accurate positioning^7^. The complex environment of rubber forests presents challenges such as obstructed GNSS signals due to leaves^8–10^, and impaired visual sensor performance in poor lighting conditions during night operation hours^11,12^. In order to cope with this complex environment, the research at the present stage of rubber-tapping robots^13–15^ mostly uses LiDAR as the primary environmental sensing hardware. LiDAR technology offers advantages like high localization accuracy, immunity to light interference, superior resolution, and robustness, making it crucial for accurate environmental sensing and positioning on rubber-tapping robots. Once environmental sensing and positioning are successfully accomplished, the rubber-tapping robot must autonomously navigate through the rubber forests. This navigation entails avoiding various obstacles, precisely advancing towards each rubber tree for operation, and adjusting its attitude to optimize the tapping process. Consequently, making correct navigation decisions and path planning play vital roles in enhancing the efficiency of the rubber-tapping robot’s navigation operations.

In previous studies, the environment perception, map construction, and accurate localization of rubber-tapping robots in rubber forests have been more mature^13^. Our focus is primarily on the implementation of autonomous navigation decision-making and path planning for these robots. Wang et al.^16^ proposed an autonomous navigation spray system for orchards that utilizes LiDAR and ultrasonic radar for environmental awareness. The system uses the center line of two rows of fruit trees detected by LiDAR as its trajectory. Upon reaching the end of a row, the system automatically turns and proceeds to the next row to continue its operations. Mao et al.^17^ introduced a navigation system for orchard harvesting robots that employs GNSS, LiDAR, and odometers for environmental sensing. This system incorporates CSF and RANSAC algorithms to determine inter-row path points. Zhang et al.^14^ developed a navigation system for a rubber-tapping robot that utilizes a 2D LiDAR and gyroscope for guidance. This system employs low-cost LiDAR and gyroscope sensors to capture sparse point cloud data from tree trunks. The point cloud data is processed using the Gauss-Newton method to fit circles around the data, allowing for the extraction of centroids for each tree. These centroids are then combined using the least-squares method to create a straight-line path, which serves as the robot’s navigational route through the forest. Fang et al.^15^ proposed an active navigation system for a rubber-tapping robot based on trunk detection. This system employs a distance-adaptive Euclidean clustering method combined with cylinder fitting and composite criterion screening to identify tree trunks. By using the tree trunks as navigation targets, the system enables the active navigation of the rubber-tapping robot.

Based on current research on robot navigation in forested areas, it is evident that many navigation methods may not perform well in irregularly planted forests and rely heavily on manual input for tasks such as turning control. In some studies, robots are merely instructed to follow predetermined navigation paths, which limits their flexibility and can lead to navigation failures when obstacles are encountered. Moreover, the autonomous navigation methods employed by a minority of robots often lack a logical sequence, negatively affecting navigation efficiency. Additionally, the rubber tapping area is determined by the initial tapping. This means that to facilitate the end effector’s operation in tapping rubber, the robot’s position and orientation must be consistently fixed each time it stops in front of the same rubber tree.

More importantly, efficient navigation in the rubber forests requires the robot to maneuver between numerous trees. A rational navigation sequence and path planning can significantly enhance the robot’s efficiency. Therefore, the key priority for enabling autonomous navigation in forest areas and advancing the natural rubber industry is to focus on developing autonomous decision capabilities for the rubber-tapping robot’s navigation and optimizing path planning procedures. Robots should be able to autonomously decide on navigation sequences, plan navigation routes, and ultimately accomplish navigation tasks in forested areas without human assistance, in line with job requirements.

In this paper, we propose a novel autonomous navigation system with a trajectory prediction-based decision mechanism for rubber forest navigation, aiming to achieve flexible autonomous navigation in rubber forest areas. This study introduces several key innovations compared to existing research:

- Robots can independently calculate and select the optimal navigation sequence without human intervention.
- After completing the navigation of a row or column, the robot can autonomously switch to the next row or column. This process aligns with practical operational requirements and significantly enhances the system’s autonomy.
- Once map construction is complete, robots can begin work from any location on the map, greatly increasing system flexibility.

The outline of this paper is as follows: In Section II, the system overview is briefly introduced, explaining the preconditions and workflow of the robot’s autonomous navigation. In Section III, a new decision mechanism for the rubber forest navigation is formulated according to the actual needs of the work and focuses on the specific components, principles, and implementation details. Finally, the experimental validation of the proposed autonomous navigation system is in Section IV. The conclusions and future work are provided in Section V.

## System overview

This paper proposes an autonomous navigation system designed for rubber-tapping robots in rubber forest areas. Built on a tracked robot chassis, the system features a trajectory prediction-based decision mechanism to facilitate autonomous navigation in these environments.

### Components of system hardware

**Fig. 1:**
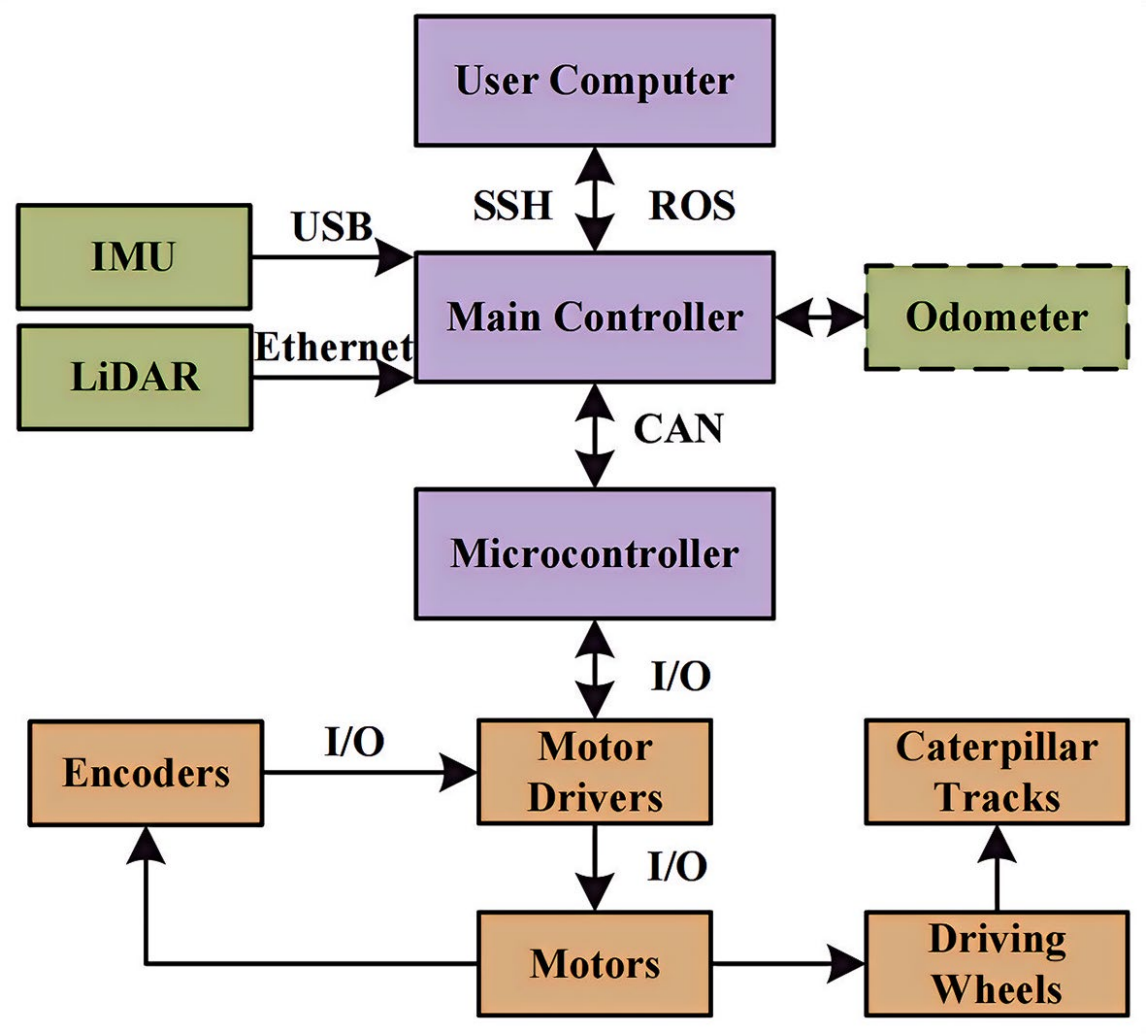
Components of the system hardware.

The hardware part of the system consists of three parts: the controller unit, the environment sensing unit, and the driving unit. The specific composition is shown in Fig. 1. The user computer, main controller, and microcontroller form the controller unit. Under the same local area network, the user computer connects to the main controller through SSH (Secure Shell)^18^ and builds a distributed remote control system using ROS (Robot Operating System)^19^ to achieve real-time monitoring and control of the robot in a longer range. LiDAR, odometer, and IMU (Inertial Measurement Unit) form an environment sensing unit, which can be used to acquire real-time information about the environment around the rubber-tapping robot and calculate the position and attitude information through the controller unit. Where the data of the odometer is received by the microcontroller from the pulse counting signal acquired by the encoder, it calculates the rotational speed of the motor, adjusts the robot’s movement speed through the PID, and sends it to the main controller to calculate and publish. Motor drivers, encoders, motors, driving wheels, and caterpillar tracks make up the drive unit.

### Preconditions of autonomous navigation

Environmental perception is a prerequisite to ensuring that rubber-tapping robots can achieve autonomous navigation in rubber forest areas by using sensors to obtain environmental information in order to construct maps of the operating area as well as robot positioning and other functions. Since the use of a single sensor results in the accumulation of errors^20^, leading to a decrease in accuracy, multiple sensors must be used to determine the same characteristics and reduce the uncertainty of the sensor^21^.

Therefore, in the navigation process of the rubber-tapping robot, it should be ensured that multiple sensors are involved in the calculation of the robot’s position during any motion. The proposed solution involves the integration of a 16-line LiDAR, wheel odometry, and an IMU, utilizing the cartographer algorithm for sensor fusion. This approach is employed either during the map construction phase to build maps or during the autonomous navigation phase to achieve precise robot localization and relocalization.

The process of sensor fusion using the cartographer algorithm differs between the map construction phase and the pure localization phase.

During the map construction phase, the cartographer algorithm first preprocesses each sensor’s data to remove noise. Subsequently, all sensor data is time-synchronized to ensure consistent location information is obtained at the same time point. The algorithm then employs an extended Kalman filter to combine odometry and IMU data and performs loop closure detection using submaps generated from LiDAR scans, thereby facilitating the construction of high-quality maps.

In contrast, during the pure localization phase, while loop closure detection is still performed using submaps generated from LiDAR scans, these submaps are no longer used for map construction. Instead, they are matched with the existing map to correct pose estimates, thereby reducing error accumulation and enabling the robot’s relocalization.

Given that IMU data can drift over time, it is necessary to correct this data using Kalman filters and complementary filters before integrating it into the multi-sensor fusion process^21^.

### Workflow of navigation system

**Fig. 2:**
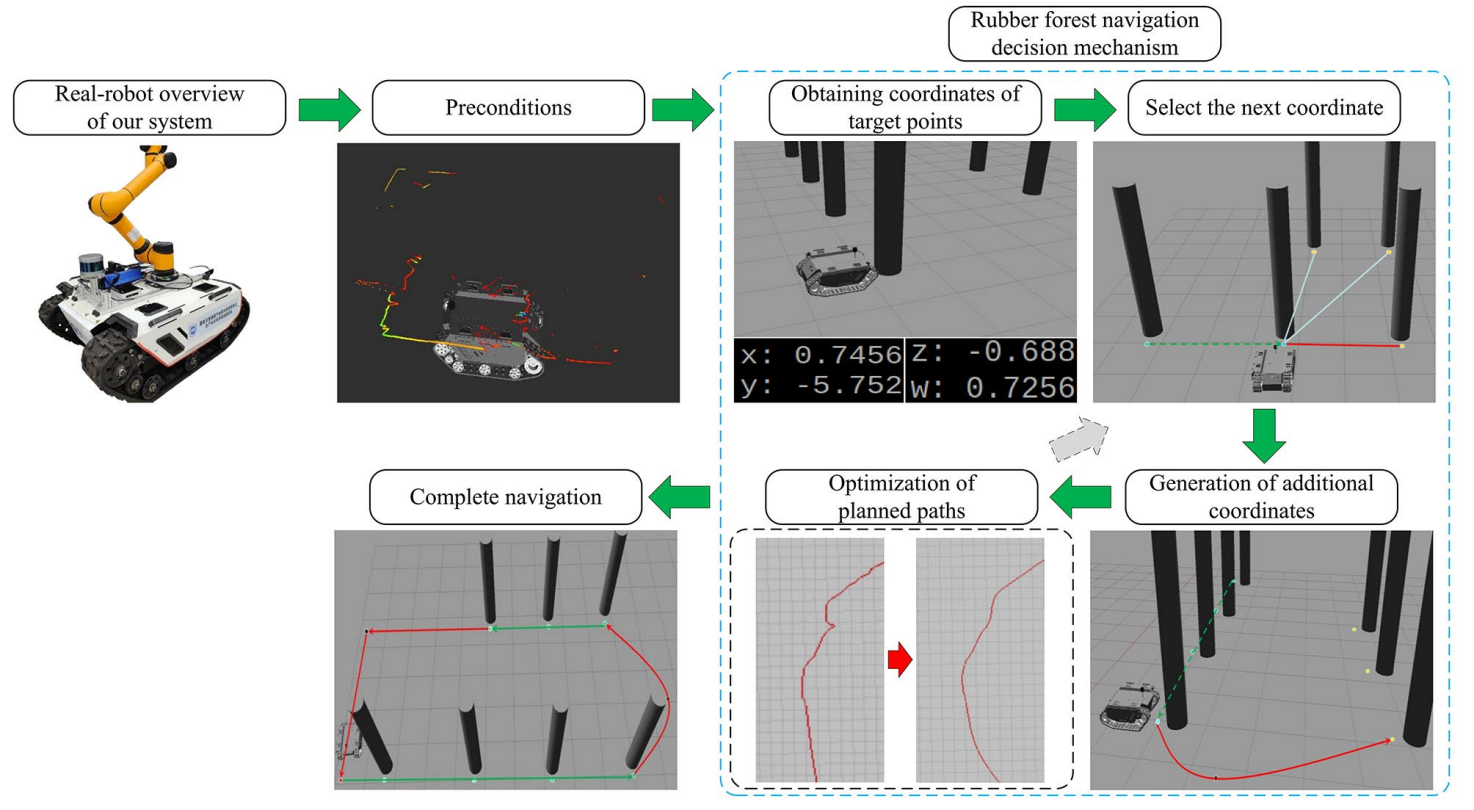
Schematic diagram of the workflow of the navigation system.

The autonomous navigation system proposed in this paper, with a trajectory prediction-based decision mechanism for rubber forest navigation, can be divided into the following these phases when working, as shown in Fig. 2.

First, the precondition for autonomous navigation is to enable the robot to have the ability to sense the environment and to perform map construction and localization by multi-sensor fusion LiDAR. Then, autonomous navigation is accomplished by the proposed navigation decision mechanism, including obtaining coordinates of target points (OCTP) on the constructed map, selecting the next coordinate (SNC), generating the additional coordinates (GAC), and optimizing the planned paths (OPP). SNC, GAC, and OPP are repeated until the navigation is completed.

It’s worth stating that mapping, coordinate recording, and orientation information recording for a rubber plantation only need to be done once because the environmental information of a rubber forest tends to remain relatively constant. This initial data collection allows for subsequent navigations without the need for repeated processes, achieving authentic autonomous navigation within the rubber forest.

## Trajectory prediction-based decision mechanism for rubber forest navigation

Due to the specificity of the rubber-tapping task, the robot needs to navigate through not just a few rubber trees, but dozens or even hundreds of rubber trees each time.

**Fig. 3:**
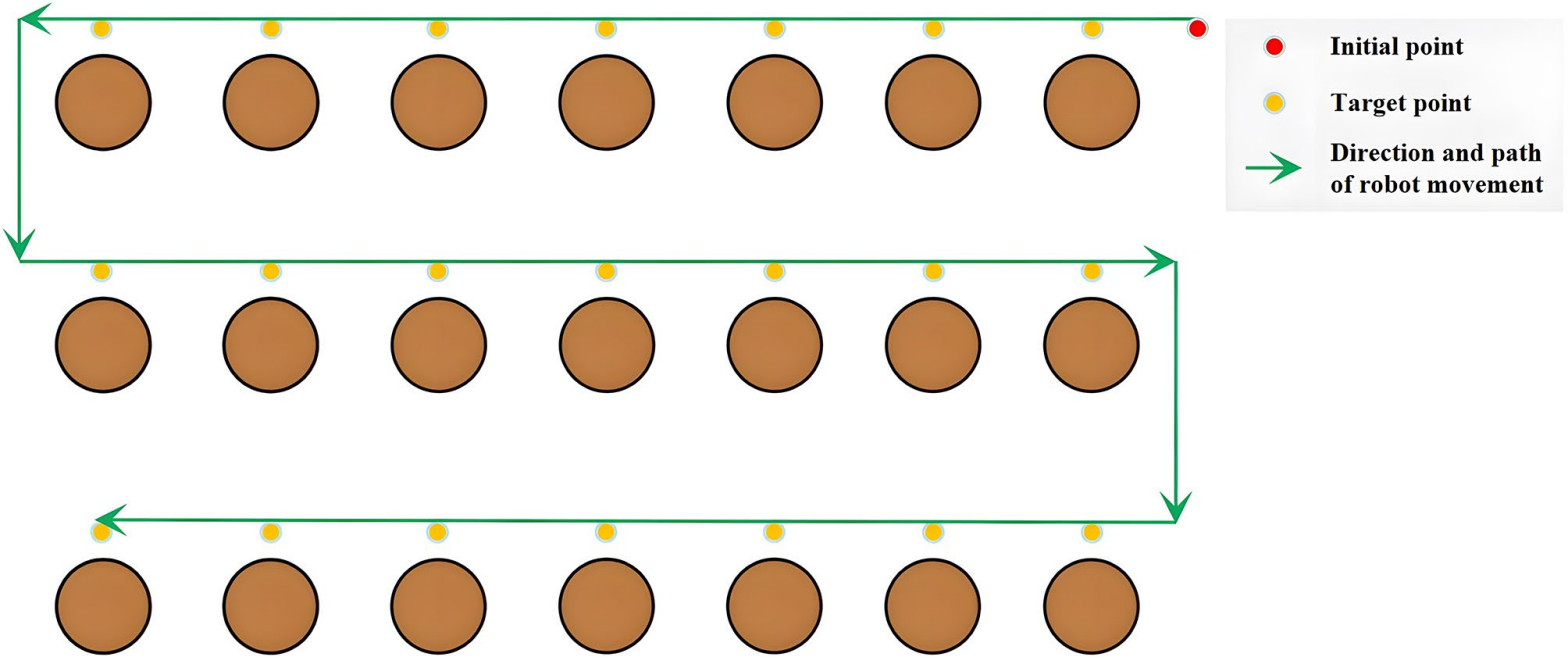
Expected robot navigation path.

In this case, the navigation sequence of many target points will greatly affect the overall navigation efficiency. Therefore, it is impossible to plan the path reasonably by using only the path planning algorithm. We need to add a navigation decision mechanism for the robot, and the expected effect is shown in Fig. 3.

For flexibility reasons, the robot will not have a fixed path manually planned by the user within each zone. The optimal navigation path should be determined by the robot itself in most instances, as it does not always start working in the same place within the zone.

Characterized by a predominant tree and weed composition, the natural rubber forest areas have a relatively structured terrain. This structured terrain facilitates efficient navigation decisions aligned with the practicalities of rubber-tapping operations, minimizing concerns about terrain factors.

The proposed program involves the following steps: First, obtain the coordinates of all designated points. Next, the robot selects the next target coordinates and determines whether additional coordinates need to be added. Then, the optimized Dijkstra algorithm is applied to generate specific paths, which are subsequently paths optimized. These paths are published to the motion control node to guide the robot’s movement, while real-time coordinates are continuously monitored and compared to the target coordinates. Once a target coordinate is reached, the next target is selected, and this process continues until all target coordinates have been reached, with the robot ultimately returning to the initial coordinate.

**Fig. 4:**
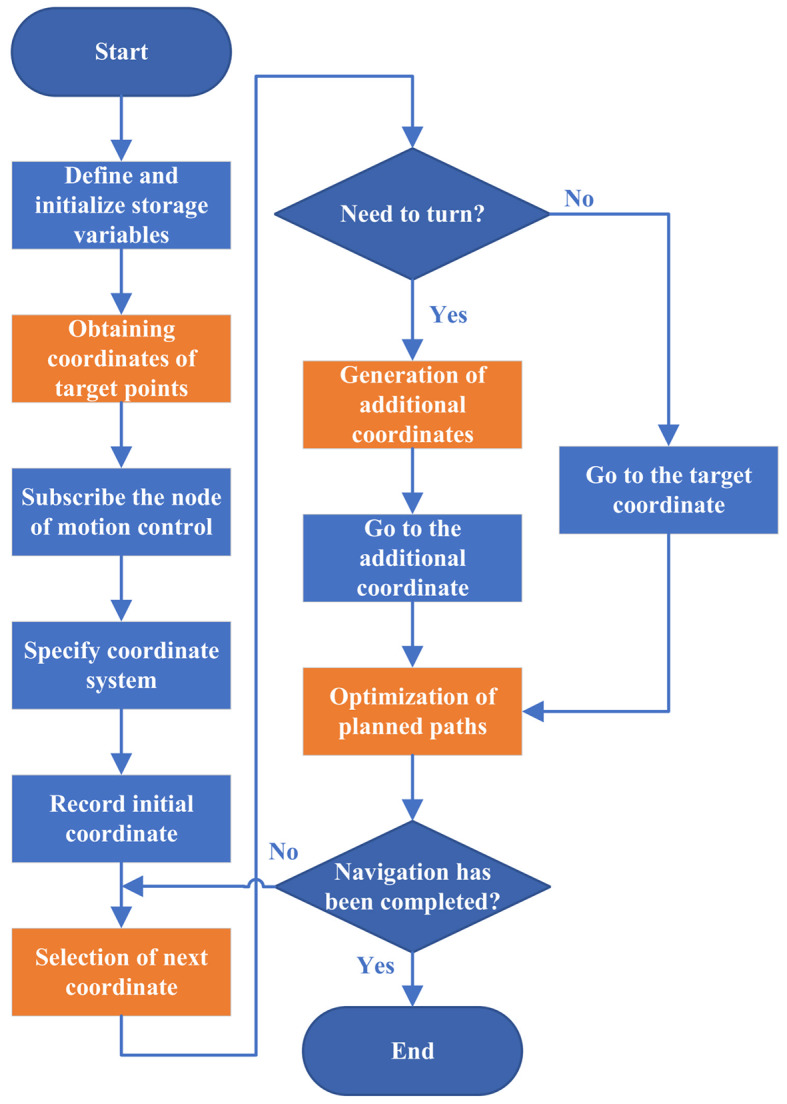
Flowchart of navigation decision mechanism.

The rubber forest navigation decision mechanism in this section mainly includes OCTP, SNC, GAC, and OPP. The flowchart of the navigation decision mechanism is shown in Fig. 4.

### Input and output

The proposed navigation decision mechanism requires the following inputs:

- Localization Information: After the mapping is completed, robot localization information is calculated and published by any localization algorithm (in ROS, this topic information is typically formatted as geometry_msgs/PoseStamped messages).

The expected outputs include:

- Navigation Path: A sequence of navigation waypoints is provided in order, along with additional coordinates necessary for the robot to switch rows or columns.
- Decision Information: Subscribe to the robot’s motion node and issue commands to control the robot’s movement along the navigation path.

For instance, when autonomous navigation begins, the robot reads the constructed map and obtains location information. After collecting the target point coordinates, it uses the proposed navigation decision mechanism to compute and generate the navigation sequence and specific path. This decision information then controls the robot to complete the navigation task.

### Obtaining Coordinates of Target Points

**Fig. 5:**
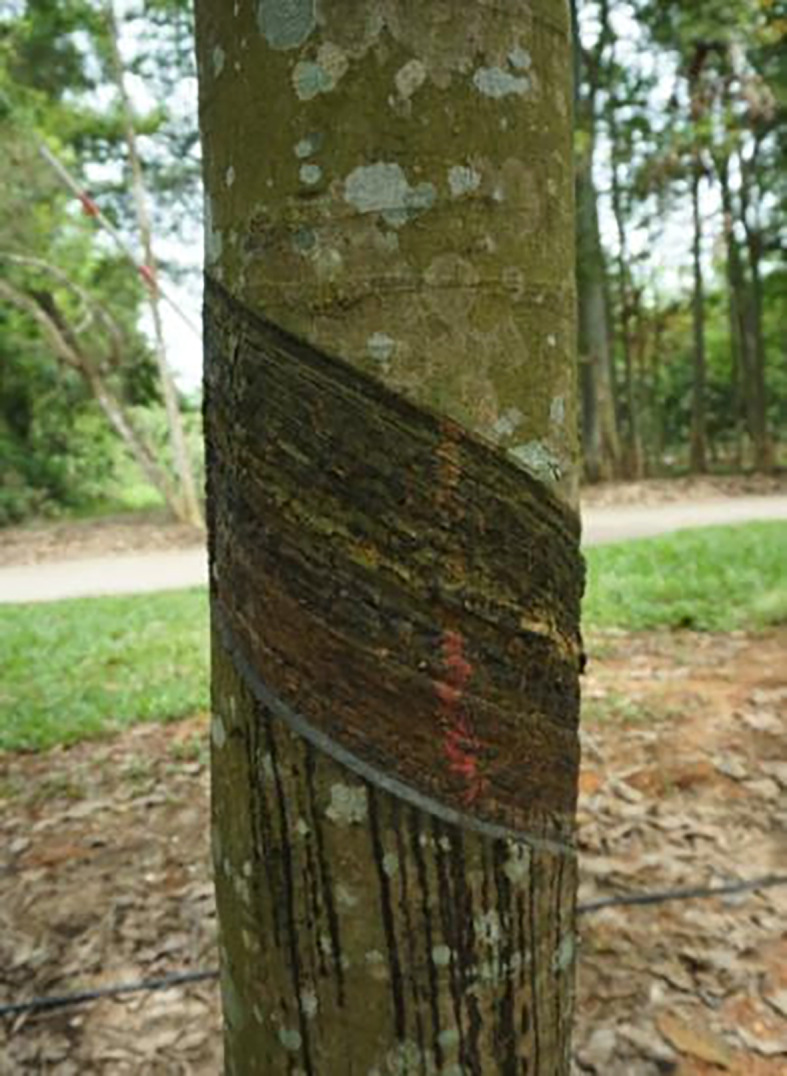
Rubber-tapping area, an area similar to a parallelogram.

The rubber-tapping operation is performed by aligning the tapping tool, such as a cutter or an end-effector, with the tapping area^2^, which is shown in Fig. 5. Therefore, in order to facilitate the rubber-tapping operation after completing the navigation task is completed, the robot should not only be parked next to the rubber tree but also in front of the tapping area of the rubber tree.

Consequently, following the conclusion of the mapping procedure described above, direct the robot to navigate to the designated rubber tree tapping location. Capture the robot’s coordinates on the generated map at this juncture and document them. This data collection is a one-time requirement. Subsequent rubber tapping activities within the same vicinity can utilize this recorded information for autonomous navigation.

The adopted approach to recording the coordinates is as follows: After the mapping work has been completed, real-time coordinates are obtained by cartographer algorithm as the robot moves across the map. The coordinates are stored in real-time as text in a file, then the text is split when reading the coordinates, converted to floating-point form, and finally stored in the array variable “*Array*”. If, for practical production reasons, the operation does not want to tap rubber from certain trees, it is sufficient to mask the coordinates of the corresponding trees.

### Selecting the next coordinate

**Fig. 6:**
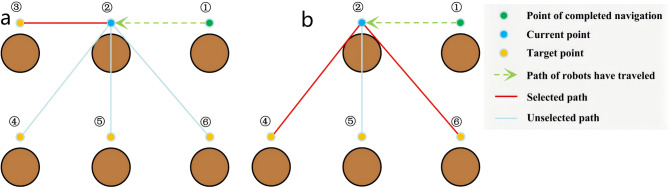
Selecting the next coordinate. (**a**) Example I. (**b**) Example II.

The objective of this stage of the algorithm is to identify the optimal coordinate within the array variable “*Array*” that contains the coordinates and subsequently transmit this information to the motion control node of the robot. This section of the algorithm involves comparing the current coordinates with the remaining tasks, performing score calculations, and ultimately choosing the coordinate with the highest score, as demonstrated in Fig. 6(a). In this figure, point 3 is the selected coordinate. This process will be iterated until the robot has traversed all the specified coordinates.

In Algorithm 1, the SNC framework is outlined. The “*Array*” variable serves as a storage for coordinate information. The “*Tree*” variable indicates the total number of target coordinates, “*DeleteTree*” represents the number of target coordinates that have been reached, and “*OtherTree*” denotes the remaining target coordinates. The class “*Goal*” is employed to store and publish the horizontal and vertical coordinate values to the motion control node. The variable “*Score*_*i*_” is used to represent the score for the i-th coordinate being processed, and “*ScoreSave*” temporarily stores this score. Additionally, the “*number*” variable records the location of the coordinates associated with the current score.


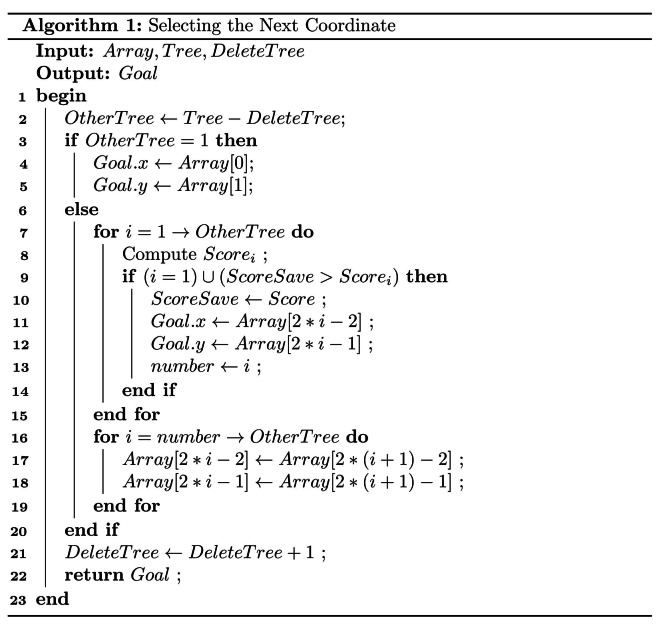


The crucial aspect of this algorithm is based on the current coordinate to evaluate the score for the remaining coordinates to be addressed, considering not only the distance between points but also obstacles such as trees. This consideration is essential because it can lead to the robot making unnecessary turns and impacting the navigation sequence. As shown in Fig. 6(b), in the pursuit of a logical navigation sequence (2-4-5-6 or 2-6-5-4), point 5 is typically not prioritized, and either point 4 or point 6 is chosen as the next target.

To calculate the score and prevent the additional coordinates generated in the inflation layer, Eq. (1) is used to represent the circular cost region corresponding to each target coordinate, with “$\Delta$” as the radius, using the center of the tree as the center of the circle. Where “$\Delta$” denotes the distance between each target coordinate and the center of the tree corresponding to that coordinate, and “*i*” denotes the *i*-th target coordinate.

1 $$\:{{\rm{P}}_{\rm{i}}}\:{\rm{ = \{ }}({\rm{x,}}\:{\rm{y}}){\rm{|}}{{\rm{x}}^{\rm{2}}}{\rm{ + }}{{\rm{y}}^{\rm{2}}}{\rm{ = }}{\Delta ^{\rm{2}}}{\rm{,}}\,{{\rm{x}}_{\rm{i}}} - \Delta < {\rm{x}} < {{\rm{x}}_{\rm{i}}}{\rm{ + }}\Delta ,\:{{\rm{y}}_{\rm{i}}}{\rm{ - }}\Delta < {\rm{y}} < {{\rm{y}}_{\rm{i}}}{\rm{ + }}\Delta \}$$

In Eq. (2), the set “*Q*” represents the cost region of all the target coordinates.

2 $$\:\:{\rm{Q}}\:{\rm{ = }}\:{{\rm{P}}_{\rm{1}}}{\rm{ + }}{{\rm{P}}_{\rm{2}}}{\rm{ + }}{{\rm{P}}_{\rm{3}}}{\rm{ + }} \ldots {\rm{ + }}{{\rm{P}}_{\rm{n}}}\:{\rm{ = }}\:\sum\limits_{i = 1}^n {{{\rm{P}}_{\rm{i}}}}$$

Next, introduce the sampling point. The distance between the current coordinate and the next coordinate to be worked on is divided equally into “$\xi$” parts. These “$\xi$” sampling points are denoted by the set “$\:{\text{J}}_{\text{i}}$” in Eq. (3), where “$\:{\text{x}}_{\text{now}}$” and “$\:{\text{y}}_{\text{now}}$” denote the horizontal and vertical values of the current coordinates, “$\:{\text{x}}_{\text{i}}$” and “$\:{\text{y}}_{\text{i}}$” denote the horizontal and vertical values of the *i*-th target coordinate. It is worth noting that the value of “$\xi$” should be set appropriately. If it is too small, there will be insufficient sampling points to detect the cost region. Conversely, if it is too large, there will be an excess of sampling points, potentially diminishing system performance.

3 $$\begin{array}{l}\:{{\rm{J}}_{\rm{i}}}\:{\rm{ = }}\:\{ ({{\rm{x}}_{{\rm{now}}}}{\rm{ + }}\frac{{{{\rm{x}}_{\rm{i}}}{\rm{-}}{{\rm{x}}_{{\rm{now}}}}}}{\xi }{\rm{,}}\:{{\rm{y}}_{{\rm{now}}}}{\rm{ + }}\frac{{{{\rm{y}}_{\rm{i}}}{\rm{-}}{{\rm{y}}_{{\rm{now}}}}}}{\xi }{\rm{),}}\:{\rm{(}}{{\rm{x}}_{{\rm{now}}}}{\rm{ + 2}} \times \:\frac{{{{\rm{x}}_{\rm{i}}}{\rm{-}}{{\rm{x}}_{{\rm{now}}}}}}{\xi }{\rm{,}}\:{{\rm{y}}_{{\rm{now}}}}{\rm{ + 2}} \times \:\frac{{{{\rm{y}}_{\rm{i}}}{\rm{-}}{{\rm{y}}_{{\rm{now}}}}}}{\xi }{\rm{),}}\:{\rm{(}}{{\rm{x}}_{{\rm{now}}}}{\rm{ + 3}} \times \:\frac{{{{\rm{x}}_{\rm{i}}}{\rm{-}}{{\rm{x}}_{{\rm{now}}}}}}{\xi }{\rm{,}}\\\:{{\rm{y}}_{{\rm{now}}}}{\rm{ + 3}} \times \:\frac{{{{\rm{y}}_{\rm{i}}}{\rm{-}}{{\rm{y}}_{{\rm{now}}}}}}{\xi }{\rm{),}}\: \ldots \:({{\rm{x}}_{{\rm{now}}}}{\rm{ + }}\xi \times \:\frac{{{{\rm{x}}_{\rm{i}}}{\rm{-}}{{\rm{x}}_{{\rm{now}}}}}}{\xi }{\rm{,}}\:{{\rm{y}}_{{\rm{now}}}}{\rm{ + }}\xi \times \:\frac{{{{\rm{y}}_{\rm{i}}}{\rm{-}}{{\rm{y}}_{{\rm{now}}}}}}{\xi }{\rm{)\} }}\end{array}$$

Then, in Eq. (4), the set “*K*” is used to represent the intersection of the set “*Q*” and the set “$\:{\text{J}}_{\text{i}}$”, which is used to select the sampling points in the cost region.

4 $$\:{\rm{K}}\:{\rm{ = }}\:{\rm{Q}}\: \cap \:{{\rm{J}}_{\rm{i}}}$$

Finally, “*score*” is used in Eq. (5) to denote the score between the current coordinate and the *i*-th target coordinate. The number of elements in the set “*K*” is denoted by “*Z*”. The weight of “*Z*” is indicated by “$\:\text{μ}$”. The score depends on the distance and the number of sampling points located within the cost region. The *i*-th target coordinate with the highest score is considered to be the coordinate of the next target point.

5 $$\:\:{\rm{Scor}}{{\rm{e}}_{\rm{i}}}\:{\rm{ = }}\:\frac{{\rm{1}}}{{\mu \times \:{\rm{Z + }}\sqrt {{{{\rm{(}}{{\rm{x}}_{\rm{i}}}{\rm{ - }}{{\rm{x}}_{{\rm{now}}}}{\rm{)}}}^{\rm{2}}}{\rm{ + (}}{{\rm{y}}_{\rm{i}}}{\rm{ - }}{{\rm{y}}_{{\rm{now}}}}{{\rm{)}}^{\rm{2}}}} }}$$

However, this method of calculating the score may have the opposite effect at some point, which we will address in the next section, GAC.

### Generating the additional coordinates

Following the successful implementation of the algorithm discussed in the preceding section, the robot can navigate in a reasonable order during the navigation process, ensuring efficient movement in the rubber forest.

Nevertheless, after the robot had finished navigating one row of rubber trees, when traveling to the next row of rubber trees, it would sometimes pass between the two trees instead of choosing to go around the outside.

This makes the navigation path unnatural. Moreover, to ensure the obstacle avoidance function is realized, it is typically essential to configure the parameters of the inflation layer in the local cost map. Setting the parameter too small may lead to the robot colliding with trees when passing through them. Conversely, setting the parameter too large may incorrectly indicate that the robot is colliding with obstacles while passing through trees, causing the robot to become stuck in its original position and unable to move. This situation could potentially impact the accuracy of path planning and navigation.

Allowing the robot to choose to go around the outside when switching rows or columns can largely circumvent the above problem.

**Fig. 7:**
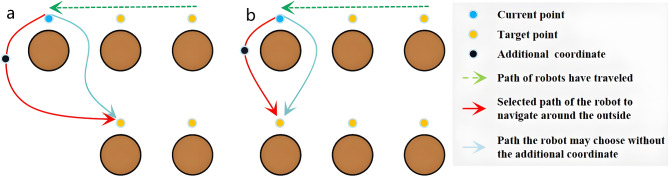
Generating the additional coordinates. (**a**) First type of row change operation (**b**) Second type of row change operation.

In point-to-point navigation, global path planning algorithms enable robots to calculate the least costly path. However, this path is rarely an outer detour, which often fails to meet our expectations when the robot switches rows or columns. To address this issue, we perform traditional trajectory prediction based on the coordinates of several navigation target points. We then add an additional coordinate to the predicted trajectory for use in outer detours during row or column changes, as illustrated in Fig. 7.

In the previous section on the SNC, the proposed score calculation may lead to some negative outcomes. For instance, as illustrated in Fig. 6(a), upon the completion of navigating point 3, the subsequent coordinate choice may favor selecting point 5 over point 4, thereby causing a disordered navigation sequence. However, through the process of the GAC, a solution emerges. Specifically, when the robot opts for point 5, it will result in the creation of an additional coordinate externally. Subsequently, upon navigating to this additional coordinate and making a new selection for the next coordinate, the score attributed to point 4 surpasses that of point 5, consequently restoring the navigation sequence to a normal progression. Furthermore, the algorithm described in this section proves effective in resolving numerous unique scenarios, though detailed enumeration of all special cases is beyond the scope of this paper.

**Fig. 8:**
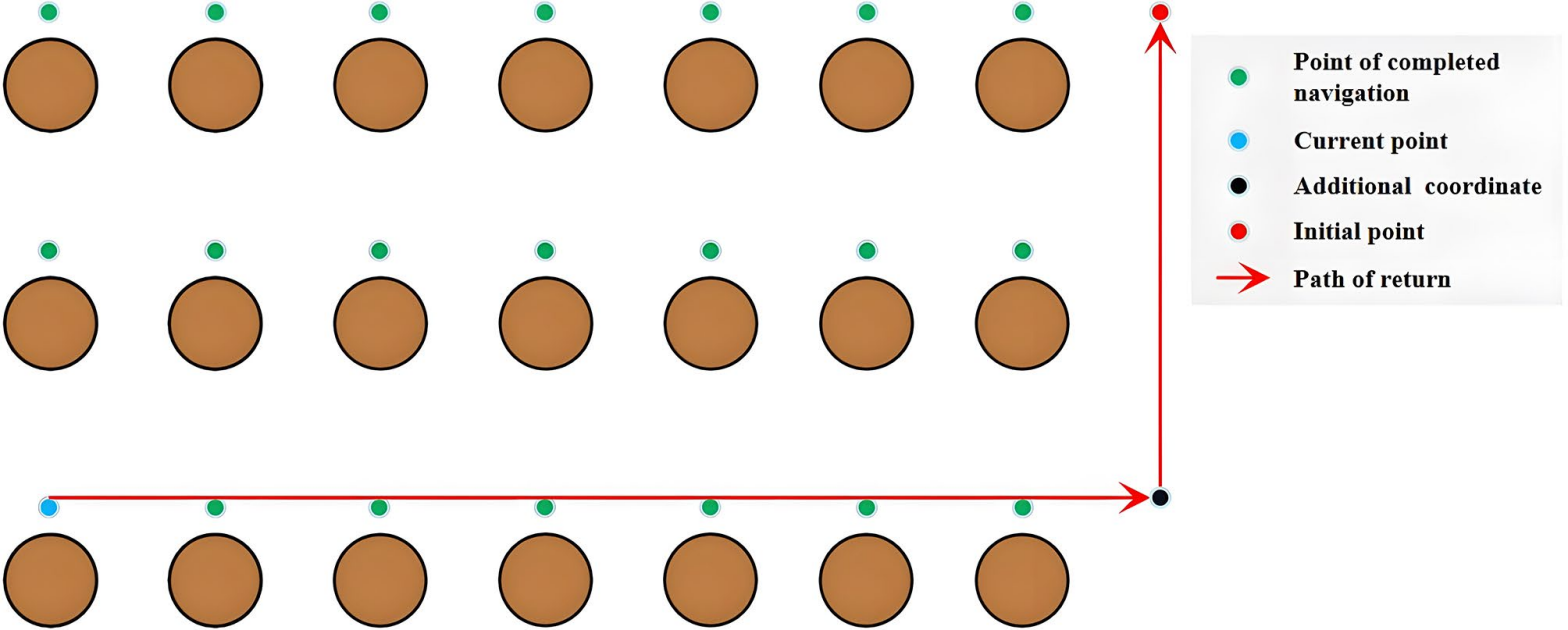
Generating the additional coordinates when returning to the initial point.

In the same way, after the robot completes navigation through all coordinates and returns to the starting point, we prefer it to bypass trees. Therefore, an additional coordinate is generated before the robot returns to the starting point, facilitating its detour, as shown in Fig. 8.

The GAC is detailed in Algorithm 2. This algorithm consists of two main parts. If a turn occurs during a line or column change, additional coordinates are predicted and inserted based on the coordinates of the current and previous points, using traditional trajectory prediction methods. If a turn occurs when returning to the initial point, additional coordinates are predicted and inserted based on the coordinates of the current and initial points. “*Ini*”, “*T*_*0*_”, “*T*_*1*_”, and “*T*_*2*_”, which are class objects used to store coordinates. “*Ini*” represents the initial point coordinates, while “*T*_*0*_”, “*T*_*1*_”, and “*T*_*2*_” represent the coordinates of the previous target point, the current target point, and the next target point, respectively. In this part of the algorithm, “*absX’*” and “*absY’*” denote the differences between the horizontal and vertical coordinates of the current target point and the previous target point, respectively. Similarly, “*absX*” and “*absY*” represent the differences between the horizontal and vertical coordinates of the next target point and the current target point. The variables “*∆x*” and “*∆y*” represent the predicted values obtained through calculation. “*Weight*_*1*_”, “*Weight*_*2*_”, “*Weight*_*3*_”, and “*Weight*_*4*_” are independent weights. The variable “*xyjudge*” is used to determine whether the robot performs a row or column change operation.


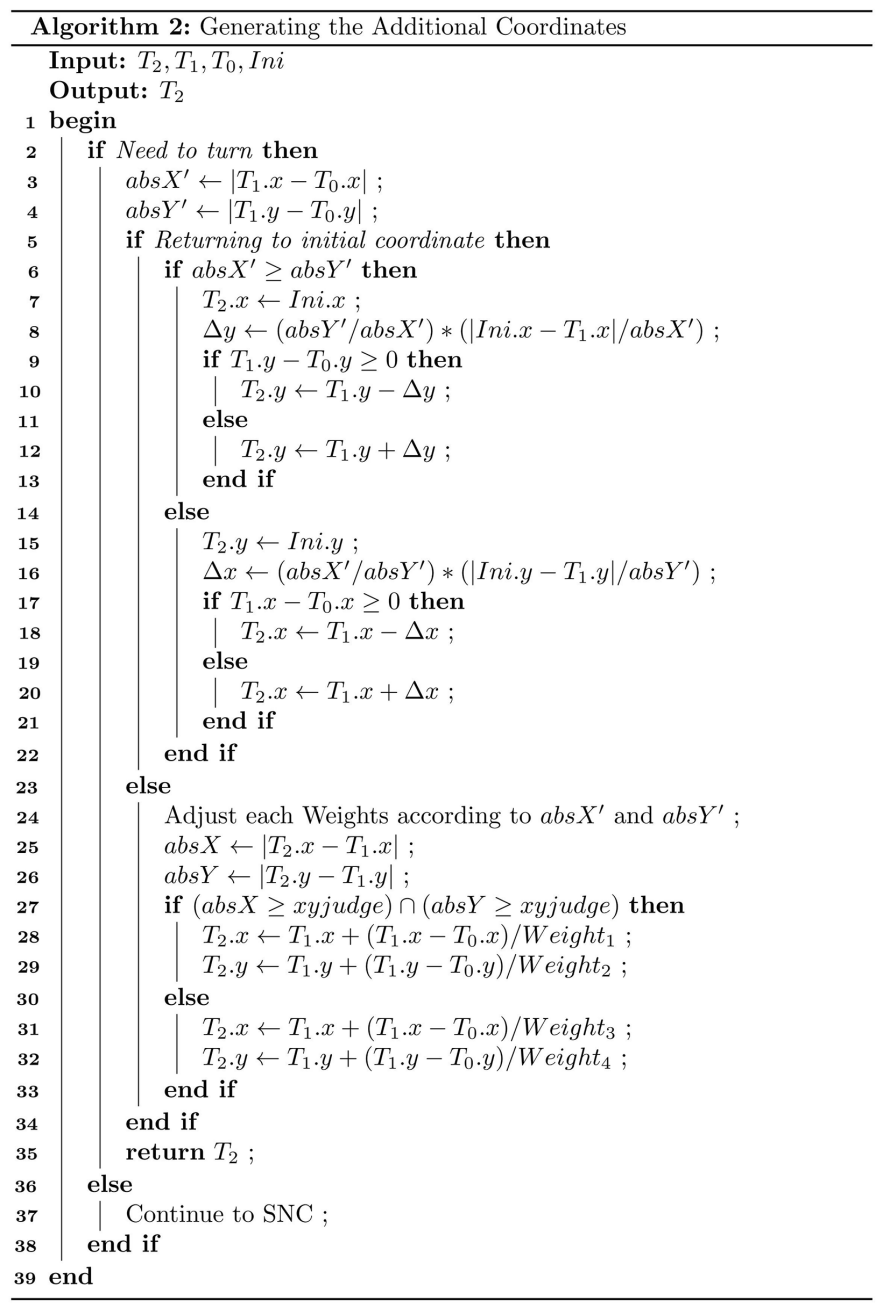


The variable “*xyjudge*” is set after constructing the map and obtaining the coordinates of the rubber trees, based on the spacing of the rows and columns of the trees. If the robot navigates by rows, “*xyjudge*” will be set to a value greater than the maximum row spacing and less than the minimum column spacing. Conversely, if the robot navigates by columns, “*xyjudge*” will be set to a value greater than the maximum column spacing and less than the minimum row spacing.

To optimize the navigation path of the robot, the values of four weights are adjusted based on the algorithm’s “*absX’*” and “*absY’*”, as these variables approximate the row and column spacing in the rubber forest. A suggested range for these values is between 2 and 5. In practice, the optimal weights for a specific work area can be identified through multiple trials.

Setting weight values for row or column change operations is crucial in navigation path planning. If these weight values are set too small, it can lead to excessive distance between the generated additional coordinates and the current coordinate, as well as the next target point, further leading to a less smooth navigation path, ultimately decreasing navigation efficiency. On the contrary, when the weight values are set too large, the additional coordinates may fall within the cost region, leading to navigation errors.

When the horizontal and vertical coordinates of the next target point significantly differ from the current position, the robot should perform the first type of row or column adjustment, using “*Weight*_*1*_” and “*Weight*_*2*_” for calculations. Conversely, if the coordinates of the next target point are similar to the current position, the robot should execute the second type of row or column adjustment, using “*Weight*_*3*_” and “*Weight*_*4*_” for calculations.

### Optimizing the planned paths

The earlier section of the study establishes the sequence of navigation and sketchy navigation routes, while specific navigation paths are generated using path planning algorithms.

Based on Fig. 3, the path planning algorithm typically requires short-distance, obstacle-free path planning most of the time. This directs our focus on selecting algorithms based on computational speed and storage space utilization. The aim is to enable the implementation of these navigation systems even on main controller with lower performance. In this context, the Dijkstra algorithm emerges as a favorable choice. Dijkstra’s algorithm holds significant popularity for determining the shortest paths between two nodes in a graph. Therefore, without delving into its underlying principles at this juncture, we will mainly introduce the improvements we made.

After applying the algorithm as a global path planning algorithm, it must be noted that the algorithm operates as a greedy algorithm, prioritizing the distance between nodes as the sole criterion for selecting the next node to expand. This approach, although efficient in terms of distance optimization, may fall short in certain scenarios due to its lack of consideration for other relevant factors. Consequently, this local optimal selection strategy can potentially result in unnecessary bends or detours along the path, leading to an overall unsmooth trajectory. Notably, the unsmoothness of the path has the implication of inducing additional rotations of the robot, thus impacting the overall efficiency and effectiveness of the navigation process.

To enhance the efficiency of the rubber-tapping robot’s movement, we optimized the path generated by the Dijkstra algorithm and forwarded it to the robot’s motion control node after completion of the optimization process.

The paths optimization algorithm is shown in Algorithm 3. In this context, “*path*” refers to the path array generated by Dijkstra’s algorithm. The variable “*AngleRange*” denotes the angle threshold, while “*SizeOfPath*” specifies the length of the path array. Additionally, “*Angle*” represents the angle formed by the coordinates of three consecutive points within the “*path*”.


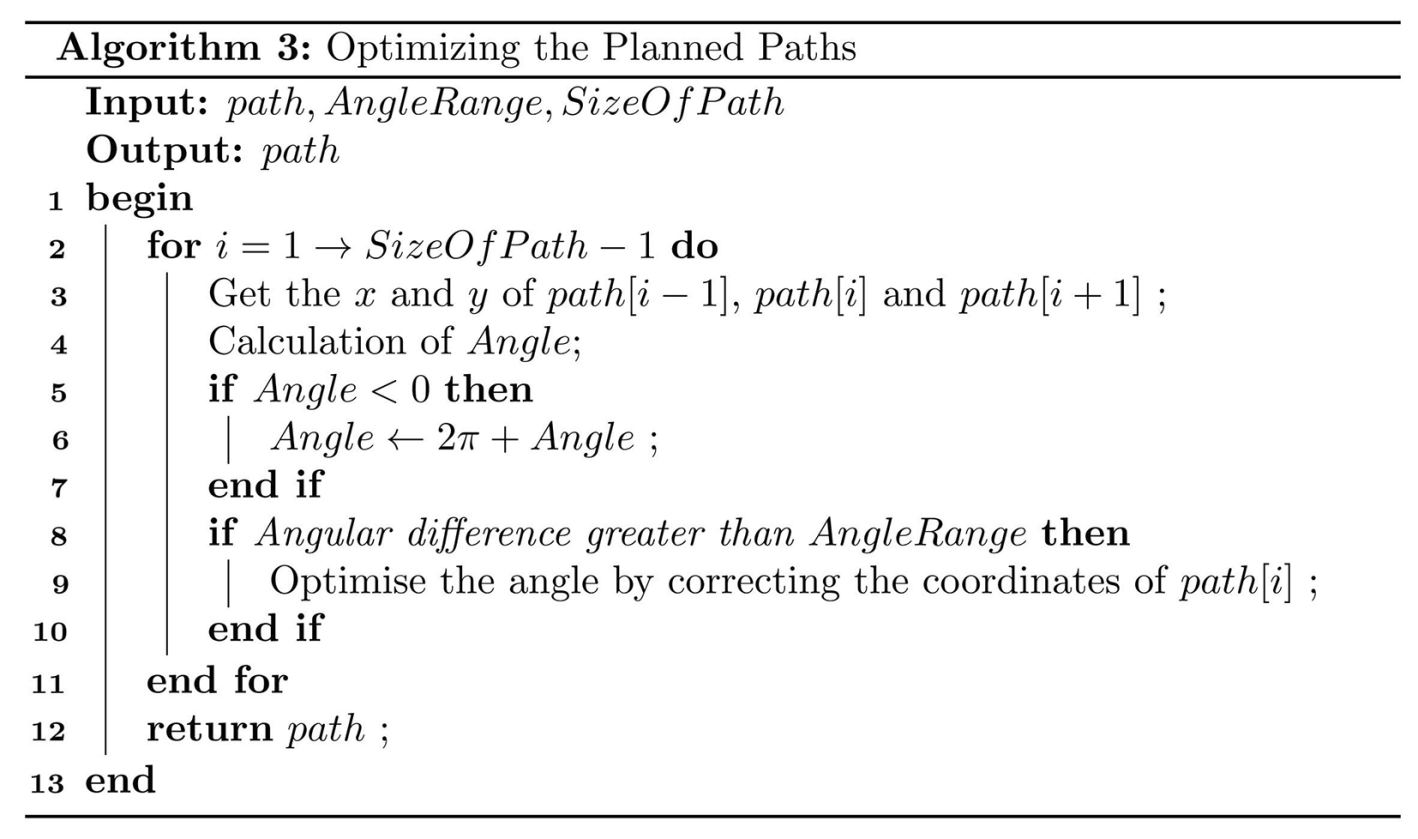


The calculation of “*Angle*” is subcategorized into Eq. (6) and Eq. (7). “*Angle*_*1*_” represents the angle between the line segment connecting the previous moment’s coordinates to the current moment’s coordinates and the horizontal line, while “*Angle*_*2*_” denotes the angle between the line segment linking the current moment’s coordinates to the next moment’s coordinates and the horizontal line.

6 $$\:\text{Angl}{\text{e}}_{\text{1}}\text{\:}\text{=}\text{\:}\text{arctan}\frac{{\text{y}}_{\text{i}}\text{-}{\text{y}}_{\text{i-1}}}{{\text{x}}_{\text{i}}\text{-}{\text{x}}_{\text{i-1}}}$$

7 $$\:\text{Angl}{\text{e}}_{\text{2}}\text{\:}\text{=}\text{\:}\text{arctan}\frac{{\text{y}}_{\text{i+1}}\text{-}{\text{y}}_{\text{i}}}{{\text{x}}_{\text{i+1}}\text{-}{\text{x}}_{\text{i}}}$$

When the difference between Angle_1_ and Angle_2_ is greater than the threshold value “*AngleRange*”, the coordinates of “*path*[*i*]” will be processed according to Eq. (8) to smooth out the path.

8 $$\:\left({\text{x}}_{\text{i}}\text{,}\text{\:}{\text{y}}_{\text{i}}\right)\text{\:}\text{=}\text{\:}\left(\frac{{\text{x}}_{\text{i-1}}\text{+}{\text{x}}_{\text{i+1}}}{\text{2}}\text{,}\text{\:}\frac{{\text{y}}_{\text{i-1}}\text{+}{\text{y}}_{\text{i+1}}}{\text{2}}\right)$$

**Fig. 9:**
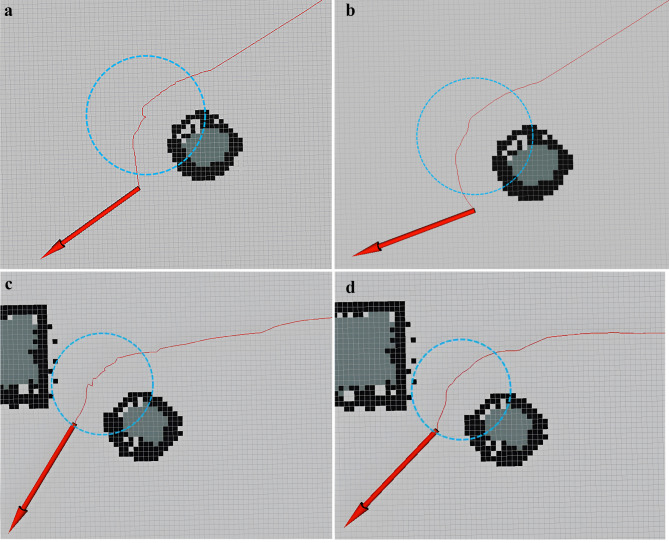
Comparison of the effect of the algorithm before and after optimization. (**a**) Example I before optimization. (**b**) Example I after optimization. (**c**) Example II before optimization. (**d**) Example II after optimization.

The result after optimization is shown in Fig. 9.

In order to enable the rubber-tapping robot to quickly adapt to real-time requirements and make adjustments in response to changes in the environment, a local path planning algorithm needs to be used. The selected algorithm for this purpose is the DWA (Dynamic Window Approaches) local path planning algorithm. Through multiple tests, the algorithm parameters are adjusted to ensure that the planned local paths meet the requirements for obstacle avoidance while aligning with the global path planned by the optimized Dijkstra algorithm. This fusion of navigational decision-making mechanisms enhances the robot’s ability to respond effectively in dynamic situations and when unexpected obstacles are encountered.

## Experimental setup

**Fig. 10:**
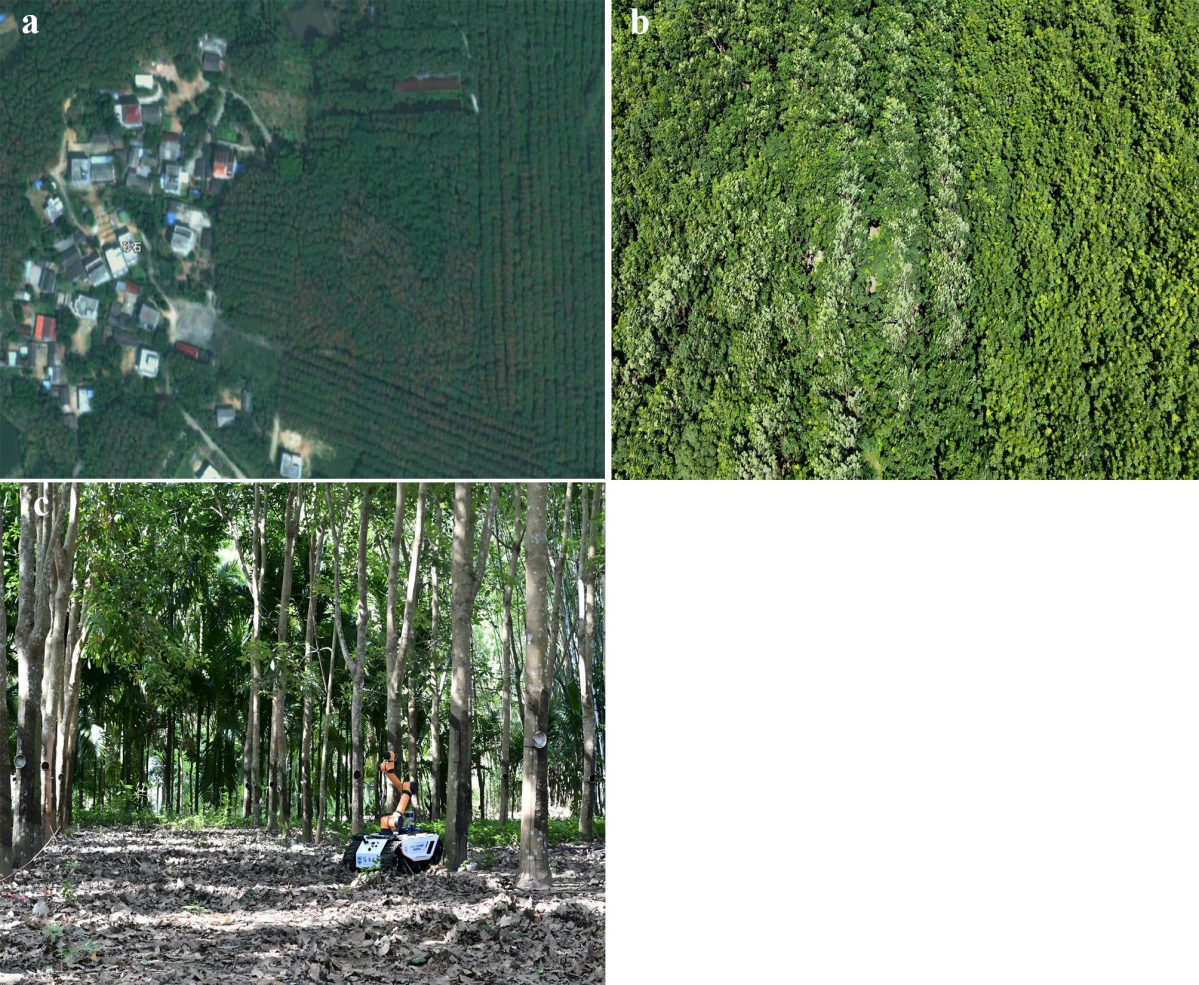
Experimental site in rubber forest area, Danzhou City, Hainan Province, China. Satellite imagery in (a) was obtained from the [China National Platform for Common GeoSpatial Information Services] (URL: https://map.tianditu.gov.cn). (**a**)Satellite map of the experimental site. (**b**) Top view of the experimental site. (**c**) Robot is operated at the experimental site.

The proposed autonomous navigation system is tested in an experimental base located in the rubber forest area of Danzhou City, Hainan Province, China. The test site, depicted in Fig. 10, is a real rubber forest area where rubber-tapping operations are conducted every three days. The site measures approximately 180 m in length and 20 m in width. In order to conduct comprehensive tests, two relatively flat areas are chosen within the site, and similar experiments are conducted in both locations.

In this experiment, we use a NUC5i3RYH mini-computer, running Ubuntu 18.04 and ROS Melodic, as the main controller. Use AgileX Bunker as the robot chassis and use the microcontroller built into the chassis to communicate with the main controller. The user computer, also running Ubuntu, connects to the main controller via SSH within the same local network to launch the ROS core, modify code using Visual Studio Code, and debug the system through the Linux terminal. Finally, visualization is achieved in RVIZ, and data such as the processing speed of related algorithms are obtained through ROS logs. The IMU used is the WHEELTEC N100 nine-axis IMU, the LiDAR is the RoboSense 16-line laser radar, and the odometry data is calculated using an incremental encoder.

Before commencing the experiment, it is necessary to configure the robot’s parameters. During the autonomous navigation phase, the submap save count is set to 3, resulting in a processing speed of 28 ms for the cartographer algorithm and 297 ms for the autonomous navigation decision algorithm. To optimize the accuracy of mapping and localization, it is essential to regulate the robot’s speed effectively. Therefore, set the maximum running speed of the rubber-tapping robot at 0.3 m/s and the maximum steering speed at 0.25 rad/s to ensure the stability of the navigation system while maintaining operational efficiency. Furthermore, to guarantee effective obstacle avoidance functionality, the inflation radius of the global cost map is defined as 0.8 m and the inflation radius of the local cost map as 0.6 m. Finally, for the purpose of preventing the robot from constantly fine-tuning when it reaches the vicinity of the target point, which affects the navigation efficiency, the determination radius is set to 0.3 m, i.e., when the robot is located within a radius of 0.3 m from the target point, it is considered that the robot reaches the target point.

After determining the motion parameters of the robot, the construction of the rubber forest map commences. In this step, one of the maps is intentionally skewed in some angle to validate the proposed GAC in Section III. The aim is to determine if additional coordinates can be added reasonably even if the rows and columns of the rubber tree do not align with the coordinate system of the map. After the robot records the coordinates of each target point on the established map and its coordinate system, the recorded coordinates are then analyzed to determine the parameters of the navigation decision mechanism.

## Experimental results

The coordinates of the target points recorded before robot navigation and the coordinates reached during autonomous navigation are shown in Tables 1 and 2. 

**Table 1:** Robot Test Data in the First Area.

Number	Target Points	Robot coordinates	Error (m)	Distance from the robotic center (m)
1	(2.16466, -1.62357)	(2.08942, -1.32617)	0.30677	0.55853
2	(5.06991, -1.71568)	(4.82057, -1.41928)	0.38733	0.53217
3	(8.12448, -1.35035)	(7.94259, -1.06229)	0.34068	0.58293
4	(11.1408, -1.3053)	(10.8485, -1.01351)	0.41301	0.55274
5	(10.9264, 3.92436)	(11.2234, 4.04123)	0.31917	0.62331
6	(7.89143, 3.69035)	(8.18361, 3.81081)	0.31604	0.59645
7	(4.94215, 3.30037)	(5.23612, 3.53417)	0.37561	0.54933
8	( 1.85719, 3.01086)	(1.63241, 2.89231)	0.25413	0.58656
9	(1.25015, 6.32553)	(1.54019, 6.30361)	0.29087	0.65622
10	(4.00741, 6.64741)	(3.98542, 6.60242)	0.05008	0.64912
11	(7.12203, 6.96602)	(6.82735, 6.86716)	0.31082	0.52791
0	(0.12332, 0.98353)	(0.33456, 1.13469)	0.25975	None

**Table 2:** Robot Test Data in the second area.

Number	Target Points	Robot coordinates	Error (m)	Distance from the robotic center (m)
1	(0.70947, 1.52478)	(0.67466, 1.35161)	0.17663	0.51433
2	(2.89545, 1.37402)	(2.83482, 1.07924)	0.30095	0.52369
3	(5.13168, 1.39915)	(4.99742, 1.10570)	0.32271	0.56572
4	(7.87044, 1.27352)	(7.64063, 1.22738)	0.23440	0.55281
5	(10.20718, 1.29865)	(9.92614, 1.22414)	0.29075	0.61353
6	(7.24330, 4.64195)	(7.05548, 4.40124)	0.30532	0.53412
7	(4.35076, 4.64045)	(4.18943, 4.41823)	0.27461	0.52974
8	(1.38789, 4.60658)	(1.66391, 4.41733)	0.33467	0.58443
9	(1.41302, 7.85660)	(1.65136, 7.57702)	0.36738	0.56320
10	(3.97589, 7.93198)	(3.86249, 7.69513)	0.26260	0.54252
11	(6.79003, 8.08274)	(6.62542, 7.88619)	0.25638	0.58115
0	(-0.06944, 3.13286)	(0.14572, 3.22473)	0.23395	None

The data in the table clearly indicates that in the first area, the average spacing of each row of the rubber tree is 2.98369 m, with a maximum value of 3.11462 m and a minimum value of 2.75726 m; the average spacing of each column is 3.95585 m, with a maximum value of 4.98021 m and a minimum value of 3.16484 m. For the sake of the stability of the navigation system and the normal operation of the navigation decision-making mechanism, the variable “*xyjudge*” is set to 3.14.

The average spacing of each row of the rubber tree in the second area is 2.66355 m, of which the maximum value is 2.96287 m and the minimum value is 2.18598 m; the average spacing of each column is 3.291545 m, of which the maximum value is 3.32745 m and the minimum value is 3.25564 m. Also, for the sake of the stability of the navigation system and the proper functioning of the navigation decision mechanism, the variable “*xyjudge*” is set to 3.1.

In this section, the evaluation of the whole system is divided into two key components. First, the focus is on the rationality of the navigation sequence and path to confirm the feasibility of the proposed navigation decision mechanism. Second, the evaluation also encompasses assessing the accuracy exhibited during autonomous navigation to affirm the reliability of the autonomous navigation system.

### Evaluate the rationality of navigation sequences and routes

**Fig. 11:**
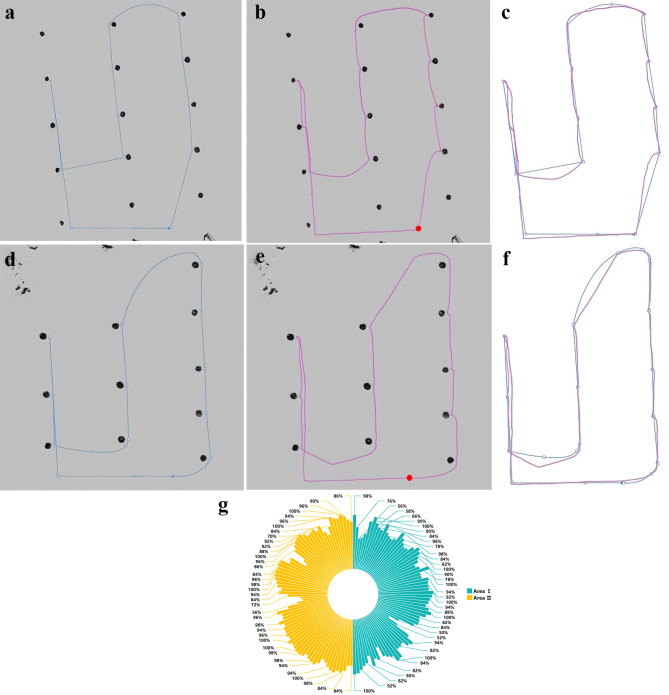
Verification of the plausibility of the planned paths. In the first area, the map construction angle is slightly tilted. (**a**) Ideal navigation path of the area I. (**b**) Actual navigation path of the area I. (**c**) The paths extracted in area I.(**d**) Ideal navigation path of the area II. (**e**) Actual navigation path of the area II. (**f**) The paths extracted in area II. (**g**) Rationalization of planned paths.

The paths and data of this navigation experiment are shown in Fig. 11. Figure 11(a) and 11(b) illustrate the ideal navigation paths, while Fig. 11(d) and 11(e) depict the actual navigation paths of the robot in the two selected areas, respectively. The ideal navigation path is a superior route determined by a detailed analysis of a 2D map drawn by the robot in conjunction with the actual situation. In contrast, the actual navigation path refers to the trajectory that the robot physically traversed during the experiment. This path is determined by acquiring real-time data generated by the cartographer algorithm, which the program then processes to convert into a trajectory. As the robot moves, this trajectory is plotted on the map in real time, providing an accurate representation of the robot’s movement. Regarding the actual trajectory of the robot, it is worth noting that the obstacle avoidance function causes the robot to deviate from the inflated area around the rubber tree after reaching the target point in front of the rubber tree. As a result, the trajectory near the target point may exhibit irregularities. This problem can be solved by decreasing the inflation radius of the cost map or by positioning the target point further away from the rubber tree. However, such adjustments may increase the risk of robots colliding with rubber trees or keep the robot too far away from the tree.

The data in Fig. 11(g) is calculated by comparing the actual paths in each of the two regions with the ideal paths by taking 70 points uniformly. It is noteworthy that in Fig. 11(c) and (f), there are significant differences between certain short segments of the actual path and the idea path due to considerations to avoid collisions when adding additional coordinates.

When the ideal path coincides with the actual path, the rationality of the planned path is considered 100%. The larger the discrepancy between the two paths, the lower the rationality. In this study, the robot was deemed to have reached the target point when it was within a 0.3-meter radius of the goal. Therefore, if the discrepancy between the selected point and the ideal path is less than 0.3 m (equivalent to 66% rationality), it is deemed within an acceptable margin of error. Out of a total of 140 points selected in the two regions, 129 points (92.14% of the total) achieved a rationality of 66% or higher.

As can be seen from Fig. 11 and the above data, the proposed autonomous navigation system almost agrees with the ideal case of manual selection in the selection of the navigation target sequence and the planning of the approximate paths and is still able to accomplish the navigation task and eventually return to the initial point in spite of the slight tilt of the created map.

### Evaluate the accuracy of autonomous navigation

During autonomous navigation, the robot records the coordinates before arriving at each rubber tree automatically. The error between the recorded coordinates and the actual robot position at the target point is calculated using Eq. (9), where (*x*_*tn*_, *y*_*tn*_) represent the coordinates of the nth target point and (*x*_*rn*_, *y*_*rn*_) represent the actual robot coordinates at that target point. Subsequently, utilizing Eq. (10), the root mean square error is determined. The variance is then computed using Eq. (11), providing further insights into the accuracy of the robot’s autonomous navigation system.

9 $$\:{\text{E}}_{\text{n}}\text{\:}\text{=}\text{\:}\sqrt{{\left({\text{x}}_{\text{tn}}\text{-}{\text{x}}_{\text{rn}}\right)}^{\text{2}}\text{+}{\left({\text{y}}_{\text{tn}}\text{-}{\text{y}}_{\text{rn}}\right)}^{\text{2}}}$$

10 $$\:{\rm{RMSE}}\:{\rm{ = }}\:\sqrt {\frac{{\rm{1}}}{{\rm{n}}}\sum\limits_{i = 1}^n {{\rm{E}}_{\rm{n}}^{\rm{2}}} }$$

11 $$\:\:{{\rm{S}}^{\rm{2}}}\:{\rm{ = }}\:\frac{{\rm{1}}}{{\rm{n}}}\sum\limits_{i = 1}^n {{{\left( {{{\rm{E}}_{\rm{i}}}{\rm{ - AE}}} \right)}^{\rm{2}}}}$$

From the data summarized in Tables 1 and 2, when the determination radius is set to 0.3 m, it is evident that the average error of the robot in reaching the target point during its autonomous navigation is 0.29103 m, with a root mean square error of *RMSE* = 0.29999 m, both of which are very close to the set determination radius. The variance of *S*^*2*^ = 0.00529568, indicates a high degree of stability in the system. These results are based on a total of 24 points, including the initial point collected in both areas.

Although this study focuses solely on the navigation of tapping robots, we included the distance from the robot’s center to the tree in our evaluation criteria, considering the future use of robotic arms for tapping tasks. The data in the table indicates that in both experiments, the maximum distance from the robotic center to the tree is 0.65622 m. This distance is less than the effective working radius of most robotic arms, thereby meeting operational requirements.

In the rubber forest environment, the proposed autonomous navigation system demonstrates satisfactory performance in terms of the average error, root mean square error, and variance. These key metrics all fall within the required accuracy parameters for operational effectiveness. Consequently, the system is adequately equipped to fulfill the navigation needs of the rubber-tapping robot, enabling it to effectively carry out the task of rubber-tapping within the rubber forest.

## Conclusion

This paper presents a trajectory prediction-based decision mechanism for rubber forest navigation, aimed at enabling structured multi-objective autonomous navigation in forest environments. Using the proposed mechanism, the robot can autonomously plan navigation routes that are specifically adapted to real-world conditions when moving from any point on the constructed map.

Based on the results of the field experiments presented in the previous section, the average localization error during autonomous navigation is 0.29103 m, and the root mean square error is 0.29999 m, both of which fall within a reasonable range given the determination radius of 0.3 m. Additionally, the variance of 0.00529568 indicates high system stability. The overall reasonableness of the autonomous navigation system’s planned path is 92.14%, confirming the system’s effectiveness in multi-objective navigation in structured forest environments.

This research offers new possibilities for developing rubber-tapping robots and benefits the natural rubber industry. It also offers valuable insights for multi-objective navigation tasks in forest and orchard environments, such as the harvesting of fruit trees like apples, mangoes, and loquats.

The proposed system is still not autonomous enough because it requires recording coordinates after mapping before it can begin navigation. Furthermore, the robot’s turning paths are not smooth enough when changing rows or columns. To address these limitations, our future objectives include further enhancing this mechanism. We aim to synchronize coordinate recording during the map construction process and simultaneously calculate data such as row and column spacing. This will enable automatic adjustments to certain variables within the navigation decision-making mechanism, thereby increasing the system’s autonomy. Additionally, multiple additional coordinates may need to be added for turning, ensuring a smoother path when the robot switches rows or columns.

## Data Availability

All data generated or analyzed during this study are included in this published article.
